# Ventral striatum supports Methylphenidate therapeutic effects on impulsive choices expressed in temporal discounting task

**DOI:** 10.1038/s41598-020-57595-6

**Published:** 2020-01-20

**Authors:** Eva Martinez, Benjamin Pasquereau, Guillaume Drui, Yosuke Saga, Élise Météreau, Léon Tremblay

**Affiliations:** 1 Institut des Sciences Cognitives Marc Jeannerod, UMR-5229 CNRS, 67 Boulevard Pinel, 69675 BRON Cedex, France; 2 Université Claude Bernard Lyon 1, 69100 Villeurbanne, France

**Keywords:** Cognitive control, Decision

## Abstract

Methylphenidate (MPH) is a dopamine transporter (DAT) inhibitor used to treat attention-deficit/hyperactivity-disorder (ADHD). ADHD patients make impulsive choices in delay discounting tasks (DDT) and MPH reduces such impulsivity, but its therapeutic site of action remains unknown. Based on the high density of DAT in the striatum, we hypothesized that the striatum, especially the ventral striatum (VS) and caudate nucleus which both encode temporal discounting, can be preferential MPH action sites. To determine whether one of these striatal territories is predominantly involved in the effect of MPH, we trained monkeys to make choices during DDT. First, consistent with clinical observations, we found an overall reduction of impulsive choices with a low dose of MPH administered via intramuscular injections, whereas we reported sedative-like effects with a higher dose. Then, using PET-imaging, we found that the therapeutic reduction of impulsive choices was associated with selective DAT occupancy of MPH in the VS. Finally, we confirmed the selective involvement of the VS in the effect of MPH by testing the animals’ impulsivity with microinjections of the drug in distinct striatal territories. Together, these results show that the therapeutic effect of MPH on impulsive decisions is mainly restricted to its action in the VS.

## Introduction

Methylphenidate (Ritalin^©^, MPH) is dopamine transporter (DAT) inhibitor used as a treatment of Attention-Deficit/Hyperactivity Disorder (ADHD), a highly prevalent, clinically heterogeneous neuropsychiatric disorder characterized by impairing levels of inattention and/or hyperactivity associated with impulsive behaviors^1,2^. Among these impulsive behaviours, ADHD patients often make impulsive choices *i.e*. they choose small immediate rewards (SIR) over larger delayed ones (LDR) more often than healthy control subjects do in delay discounting tasks (DDT)^3,4^. Delay discounting paradigms are designed using the concept of temporal discounting, based on the observation that humans and animals devaluate future outcomes^5^. Impulsive choices, which derive from high discounting, are a central aspect of ADHD and delay aversion is considered as an important component in the development of this pathology^6,7^.

MPH, the primary medication used to treat ADHD, significantly improves the behavioural symptoms associated with ADHD^8^. Specifically, MPH decreases impulsive choices in ADHD patients performing DDT, in which they have to choose between an SIR and an LDR^9–11^ and also reduces discounting in healthy humans^12^, rodents^13^ and monkeys^14^. The efficacy of this treatment depends on the dose. Slezak *et al*., have shown that trial omissions increased along with dose in rats^15^ and the same effect has been reported in monkeys that became drowsy at high doses and were inconsistent in performing the task^16^. According to literature review conducted by Konrad-Bindl, *et al*. (2016), drowsiness is the most commonly recorded side effect in humans (found in up to 32% of patients)^17^. Thus, dosage of this dopaminergic agent is a central element to be considered.

At the pharmacological level, MPH blocks dopamine reuptake, enhancing dopamine signalling in the cortico-striatal circuitry^11,16,18^. However, the mechanisms and specific sites behind its therapeutic effects remain unknown. Positron Emission Tomography (PET) imaging in healthy subjects and ADHD patients has revealed that MPH increases the synaptic concentration of dopamine mostly inside the anterior striatum^16,19–21^. The striatum, which is subdivided into three territories – the Ventral Striatum, (VS), the Caudate nucleus (CdN) and the Putamen – is the structure containing the highest density of DAT which MPH acts on to increase the dopamine level^20^. Thus, it has been hypothesized that interactions between striatal subterritories and the frontal cortex may be involved in the various types of impulsivity, motor and cognitive impulsivity being mediated by distinct cortico-striatal loops^22^.

Moreover, neuronal recordings in monkeys^23^ and functional brain imaging studies in humans^24^ have shown that the VS and the CdN both encode temporal discounting, and therefore can be involved in decisional impulsivity processes. The VS is connected to the limbic system and plays a role in motivation and reward expectation. Volkow *et al*. showed that MPH induced increased dopamine release in this striatal subterritory^11^, supporting this first hypothesis. Regarding the CdN, Hong *et al*. showed that ADHD patients have a reduced CdN functional connectivity^5^, suggesting this striatal territory is involved in impulsive choices. The CdN is anatomically connected to the lateral and medial prefrontal cortex, and is involved in decision-making processes and attention, which makes it a good candidate. This second hypothesis is strengthened by our recent work, demonstrating that the CdN is involved in impulsive choices triggered by a D_2/3_ receptors agonist (pramipexole)^25^.

This study aimed to determine VS and CdN involvement in therapeutic effects triggered by MPH, taking into account the dose effect. First, monkeys were trained to perform DDT, and their impulsive state was assessed. MPH was administered intramuscularly at two doses to establish the therapeutic dose and decrease impulsive choices in healthy monkeys. Then, to visualize the location of MPH fixation at these two doses, PET scans were performed using [^11^C]-PE2I, a DAT ligand, as a radiotracer^4^. Finally, to investigate the specific role of the VS and the CdN in the therapeutic effects of MPH on impulsive choices, micro-injections of MPH were performed directly inside these two structures.

## Materials and Methods

### Animals and surgical procedure

Five monkeys were used in the study: a female *Macaca mulatta* (monkey T, 5 kg) and 4 males *Macaca fascicularis* (monkeys K, A, L and C, 6 kg). Animal care and housing were in compliant with the NIH guidelines (1996) and with the European Communities Council Directive of 2010 (2010/63/UE) recommendations. Procedures were approved by the French National Committee (#991-2015063017055778). PET imaging was performed on monkey A, L and C; behavioural task combined with intramuscular injections was performed on monkey A, K and T and intrastriatal micro-injections was performed on monkey K and T.

During the behavioural experiment, animas were seated in a primate chair and trained to perform the task. After eight months of training, a plastic chamber and head holder were fixed to the monkey’s skull under general anaesthesia and sterile conditions. Positioning of the chamber was estimated using structural MRI scans (1.5T; CERMEP, France). The centre of the MRI-compatible chamber was aligned based on the anterior commissure (AC) to allow penetration into the right anterior striatum. Detailed descriptions can be found in our previous work^26,27^.

### Apparatus and delay discounting task

During experimental sessions, a monitor equipped with a touch-sensitive screen was placed in front of the monkey and an infrared-sensitive resting key was installed on the primate chair on which the monkey kept its left hand to run the task. Presentation^©^ software (Version 18.0, Neurobehavioral Systems, Inc., Berkeley, CA) and Scenario Manager software (ISCMJ, Bron, France) controlled the successive presentation of visual cues displayed on the screen, monitored behavioural responses (screen touches), and regulated reward delivery timing. Single drops of apple juice (0.12 or 0.28 mL) were delivered via a sipper tube attached to a computer-controlled solenoid valve for successful trials.

**Figure 1:**
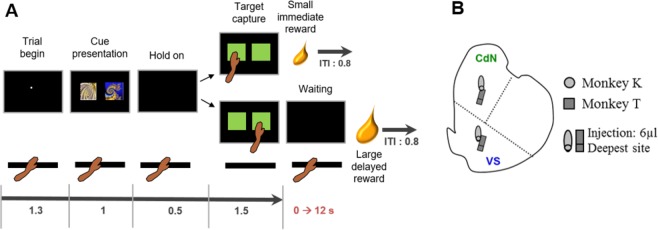
Delay discounting task and location of micro-injections. (**A**) Timeline of the instrumental paradigm in which each animal was required to make choices between a small immediate reward and a larger delayed one. A trial began when a small white dot appeared at the center of the screen and the monkey held the resting key with its left hand. Then, two peripheral cues were presented on the monitor. One of them was a conditioned stimulus associated with a small immediate reward, whereas the other cue indicated a larger delayed reward. Six different visual cues per animal were used for different delays set between 0 and 12 seconds. After offset of the peripheral cues, two green squares were displayed (the go signal), thereby cueing the animal to release the resting key and to select one of the targets. Depending on the chosen target, fruit juice was then delivered after the selected reward delay period (small immediate reward vs. large delayed one). Independent of animal choices (immediate or delayed reward after the target touching), all trials were followed by a 0.8-s inter-trial interval (ITI). (**B**) Schematic map of intra-striatal microinjection sites in the right caudate nucleus (CdN), and the right ventral striatum (VS). The sites were fairly close between the animals.

In each DDT trial (Fig. 1A), the animal was required to make a choice between an SIR and an LDR. When the monkey held the resting key with its left hand, a trial began with a small white dot appearing at the centre of the screen. After 1.3 s, two peripheral cues were presented on the screen (1 s in duration). One was a conditioned stimulus associated with an SIR (unique volume/delay combination: 0.12 mL and no delay) whereas the other indicated an LDR. Six different visual cues were used per animal for different combinations: 0.28 mL given after 0, 1.5, 3, 4.5, 6, and 9 (monkey A) or 12 s (monkeys T-K). Cue positions were randomly modified across trials to avoid a possible directional bias. 0.5 s after cue offset, two green squares appeared in the same two positions, cueing the animal to touch one of the targets (within <1.5 s). Prior to the experimental period, the animal learned the reward value (volume/delay) associated with each conditioned stimulus (visual images using fractal geometry) during a training period, and was then free to choose any option based on its preference. Depending on the chosen target, fruit juice was delivered after the selected reward delay period (short or long delay). All trials were separated by a 0.8-s inter-trial interval. Each choice combination (small vs. large reward) was repeated 30 times in a given block of trials, and the six possible blocks were presented in pseudo-random order across the session by ensuring there was no immediate repetition of the same block. To minimize the day-to-day variations in an animal’s performance due to a change in its motivational state, each monkey performed the task with a constant number of blocks (A: 14 blocks, K: 22 blocks, T: 28 blocks). Because we used constant numbers of blocks, the optimal strategy to maximize reward intake in terms of volume was always to select the LDR. Alternatively, a preference for the SIR reflected an overall delay aversion, cumulating the sensitivity to the delay occurring before the reward delivery and the total duration of the task (choosing SIR minimized the task length, while it also increased the rate of reinforcement).

Errors in task performance were categorized as follows: 1) Premature response occurred when the monkeys were unable to keep their hand on the resting key before target presentation and 2) Non-initiated choice (omission) occurred when the monkeys did not initiate a response during the 2-s target presentation. When premature responses appeared during cue presentation, the cues were removed and the current trial was stopped, followed by an inter-trial interval.

### Methylphenidate administration

Intramuscular MPH injections (0.1 and 0.6 mg/kg) were first performed five minutes before the animal started to perform the DDT. Two dose levels were tested, as wide variability in side effects has been reported depending on the dose^17^. MPH was administered once a week and control days were defined as behavioural sessions conducted one day before and two days after the testing day. This procedure was used with monkeys A, K and T.

Intracerebral MPH micro-injections were then performed on monkeys T and K (6 µL; 0.6 µg/µL) in the right anterior striatum, inside the VS and the CdN at the beginning of different sessions (Fig. 1B). Each striatal territory was tested during four sessions per monkey, and the microinjections were repeated twice weekly. The anatomic locations were determined using MRI scans and electrophysiological mapping performed with penetrations spaced 1 mm apart. Detailed descriptions of the micro-injection procedure can be found in our previous studies^26–28^.

### Analysis of behavioural data

In the model used to analyse the animals’ choices, probabilities that the monkey would choose the SIR over the LDR were calculated for each block of trials. A fitting logistic function was plotted using an exponential function to estimate the temporal discounting behaviour. The exponential discount model was found to provide a better fit for our monkeys’ behaviours than the hyperbolic discount function (the models using were compared using Akaike’s Information Criteria). The temporally discounted value (DV) was therefore calculated as follows:

$${\rm{DV}}={\rm{A}}\,\exp \,(\,-\,k{\rm{D}}),$$

where A is the volume of the reward, D is the length of the delay and *k* is the steepness of the discount function^29^. The model parameters (discount factor *k* (s^−1^) and the temperature parameter of the logistic function) were simultaneously estimated using a maximum likelihood procedure^23,30,31^. Larger *k* values indicated more impulsive choices, while smaller *k* values indicated more patience^32–34^. Based on the estimated logistic curve, the indifference point, the delay at which the animal indifferently chose the SIR or the LDR, was also calculated to characterize the temporal discounting. The lower this point, the more sensitive the animal is to delay and thus impulsive. All of the data analyses were performed using custom scripts in MATLAB.

Impulsivity markers (*k* values and indifference points) were compared between conditions (control vs. MPH days) and task periods (blocks were categorized into three equivalent temporal segments) using two-way ANOVAs. Then, local effects of intra-striatal MPH micro-injections were investigated considering only the early period in each behavioural session (i.e, unspecific effects due to drug diffusions were controlled for). Reaction times (RT; intervals between cues appearance and key release), movement times (MT; intervals between key release and target capture), and error rates were tested across drug conditions using a Mann-Whitney U-test. Non-parametric tests were used as the data sets were not normally distributed (Kolmogorov–Smirnov test, *p* > 0.05).

### PET imaging

To determine where MPH action is most prominent when the drug is administered *via* intramuscular injections (0.1 or 0.6 mg/kg), PET scans performed with a specific radiotracer for DAT (i.e. the [^11^C]-PE2I) coupled with or without injections were compared. By calculating the difference in DAT-binding potential (BP) between conditions (control-injection), DAT occupancy by the MPH was located. The MPH injection was performed 10 minutes before the [^11^C]-PE2I injection and the beginning of the acquisition. Three animals (monkeys A, L and C) were used for this procedure.

All PETs were performed at the imaging centre (CERMEP, Lyon) as described in Ballanger *et al*.^35^ and Beaudoin-Gobert *et al*.^36^. Before acquisition, each monkey was pre-treated with intramuscular injections of Atropine (0.05 mg/kg) followed by Zoletil (15 mg/kg). The animals were continuously perfused with a Ringer’s lactate solution *via* a vein catheter. A Siemens Biograph mCT/S64 scanner with a spatial transverse resolution of 4.4 mm was used. Attenuation was obtained using a 1-min low- dose CT scan acquired before emission. Images were reconstructed using the Siemens ultraHD PET algorithm with 12 iterations, 8 subsets and a zoom factor of 21. The reconstructed volumes were 109 slices (2.027 mm thickness, 256 × 256 matrices of 0.398 × 0.398 mm² voxels). PET scan acquisitions began with the intravenous injection of [^11^C]-PE2I synthesized in the cyclotron unit at CERMEP.

Regions of interest were specified via propagation of the *M. fascicularis* maximum probability atlas using the MAXPROB method^35^, with a focus on 6 regions (VS, anterior CdN, anterior Putamen, posterior ventral Putamen, posterior CdN and posterior Putamen). For each monkey, an individual MRI was saved to the *fascicularis* MRI template and each PET image was saved to the control PET image which was in turn saved to the individual MRI. Concatenation enabled direct affine transformation from PET images to template space.

## Results

### Delay discounting behaviour in monkeys

To determine individual temporal discounting and impulsive choices, three monkeys were trained to perform the DDT (Fig. 1A), in which they freely choose between a fixed SIR and LDRs. All three monkeys chose the option yielding the larger gain when both rewards were offered immediately or after a short delay. However, as the waiting time to obtain the larger reward increased, the monkeys alternatively preferred the SIR. This shift in preference reflects that the three animals considered both reward volume and delay to estimate subjective values and make their choice. Based on each behaviourally derived preference curve, a discount function was estimated to assess how subjective value declined with delay. Consistent with previous findings in a range of species such as rats^37^, macaques^38^ and humans^39^, the discount curves were characterized by exponential and hyperbolic functions. Although both models closely approximated the animals’ task performance for the majority of behavioural sessions, the exponential discount function was observed to provide the better fit in 85% of the cases (medians ± sem: AIC_exp_ = 306 ± 25 vs. median AIC_hyp_ = 311 ± 26). Hence, only data obtained with the exponential discount function are reported in this study. A median *k* discount factor of 0.22, 0.17 and 0.11 s^−1^ was calculated for monkeys A, K and T, respectively. Based on this, monkey A was therefore the most impulsive animal in normal control conditions (high *k* value), while monkey T was the most patient (low *k* value). Monkeys showed stable discount rates across sessions and *k* values were equivalent to those reported in previous monkey studies using the DDT^23,30,31,40^. The estimated discount rates matched the indifference points calculated based on the decision curve and were anti-correlated with the *k* discount factors (Spearman rho = − 0.86; *P* < 0.001), reinforcing the view that both parameters are informative in assessing impulsive decisions^41,42^. Of interest, the monkeys’ choices and temporal discounting were found to be slowly modified by the accumulation of trials through each session (*F* > 3, *P* < 0.05, one-way ANOVA). This slow effect which likely reflected a decline in the animals’ motivational state throughout a daily experimental session was controlled for in our further analysis.

### Low methylphenidate administration reduces impulsive choices

**Figure 2:**
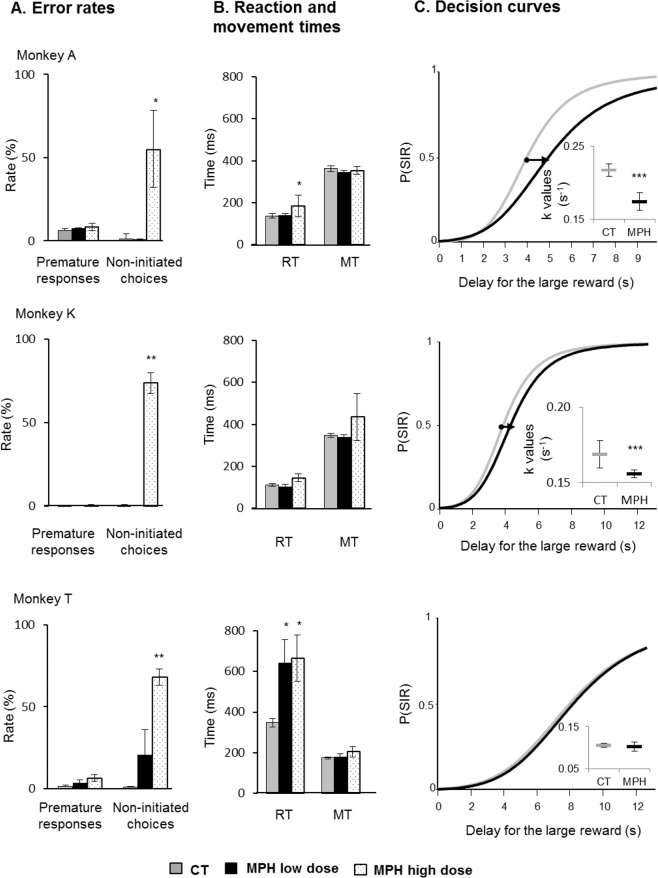
Delay discounting behaviours with intramuscular administration of methylphenidate (MPH). (**A,B**) Measures of task performance were averaged (mean ± SEM) across early periods of sessions after MPH intramuscular injections at two doses. (**A**) Non-initiated choices increased at high dose in all monkeys, and (**B**) reaction times were increased by low-dose MPH for monkey T and by high dose MPH for monkeys A and T, while there was no effect on movement times (Mann-Whitney U-test; *P < 0.05, **P < 0.01). (**C**) For each monkey, four distinct experimental sessions with an MPH injection (*black*) were compared to control days (n = 8; *grey*). Following MPH injections, there was a significant decrease in discount factor *k* for monkeys A and K (two-way ANOVA; ***P < 0.001), while no changes were detected for monkey T (*P* = 0.2). The inserts show median *k* values ± SEM. The black arrows between decision curves indicate the shift in indifference points (i.e., equal preference between reward/delay conditions) induced by the MPH.

While the three animals were performing the DDT, the systemic effects of MPH administration on the animals’ choices were tested by performing intramuscular injections (Fig. 2). As a wide variability of side effects has been reported depending on the treatment^17^, two different MPH dose levels (low vs. high dose) were tested. For each monkey, four distinct experimental sessions with a dose of MPH were compared to control days (n = 8). As shown in Fig. 2A, high-dose MPH was found to induce major impairments in the animals’ performance, with a rise in error rates during task execution (*P* < 0.05, Mann-Whitney U-test). In particular, the monkeys increased non-initiated choices by omitting their instrumental response in over half of the trials (A: 55%, K: 74%, and T: 68%) due to drowsiness induced by the drug. Despite this great effect on alertness, no major change in motor performance was observed when the animals executed the trials well (Fig. 2B). However, the limited number of choices recorded per session made it impossible to accurately estimate decision curves when high-dose MPH was administered. On the other hand, we found that low-dose MPH had an effect on temporal discounting. Of the three animals tested, two were qualified as being less impulsive with the drug injections (drug x periods, two-way ANOVA). Figure 2C shows the decision curves obtained for the early experimental period, i.e. when the drug effect was most powerful at the beginning of the session (*post hoc* Tukey-Kramer comparison). Following low-dose MPH administrations, monkeys A and K showed a decrease of 20% and 8% in their *k* values, respectively (*F* > 17, *P* < 0.001). The more this discount rate decreased, the less sensitive to delay and impulsive each monkey became. These modifications in time discounting were coupled with a shift in indifference points (*F* > 7, *P* < 0.01), in which equal preferences were increased by 0.9 (A) and 0.2 (K) seconds. No clear drug effect was found on the choices made by monkey T (*F*_(1,4)_ = 1.53, *P* = 0.2), whereas slower RTs were measured (*P < *0.05, Mann-Whitney U-test) following the MPH injections.

Overall, while high-dose MPH rendered the monkeys drowsy and unable to perform the task well, low-dose MPH made them less impulsive in their choices, as evidenced by a rightward shift in their decision curves due to a lower sensitivity to delay. Because the MPH administration method was systemic with the intramuscular injections, these different behavioural effects could emerge from different sites of action reached by the drug.

### Preponderant action of low methylphenidate administration in the ventral striatum

**Figure 3:**
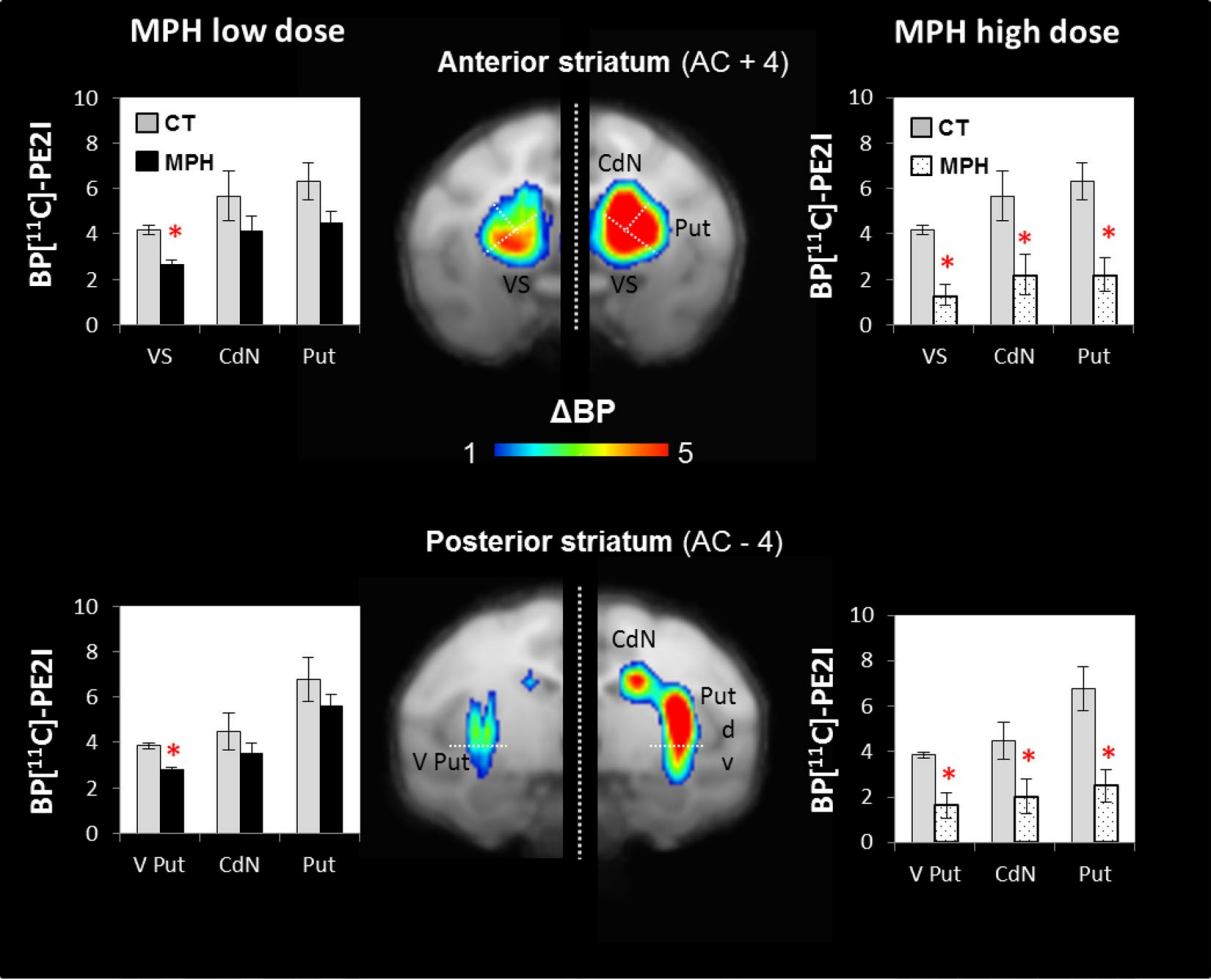
PET scan results. Histograms indicate the mean ± SEM of [^11^C]-PE2I Binding Potential (BP) of 3 monkeys, calculated based on control condition and after MPH injections. To visualize the effect of the MPH at two doses, we calculated the difference in [^11^C]-PE2I binding potential (ΔBP = BP_control_ − BP_test_) between a control condition and when it was paired with an MPH injection. At low dose, MPH decreased [^11^C]-PE2I BP in the anterior VS and the posterior ventral Putamen. At high dose, MPH decreased [^11^C]-PE2I BP in all striatal territories (Paired t-test; *P < 0.05). AC = anterior commissure; VS = ventral striatum; CdN = caudate nucleus; Put = putamen, V Put = ventral putamen; d = dorsal.

To determine where MPH action is most prominent when the drug is administered *via* intramuscular injections (low vs. high dose), PET scans performed with [^11^C]-PE2I coupled with (test) or without (control) injections were compared. Specifically, by calculating the difference in DAT-binding potential (BP) between conditions (control - injection), the territories where DAT occupancy was broadly affected by the drug were located. A high differential BP value indicated that MPH strongly influenced DAT binding, while a low differential value reflected no interaction with the binding. Figure 3 illustrates the population-averaging results obtained using the differential imaging of three monkeys (A, L and C). With low-dose MPH (previously defined as therapeutic on impulsive choice), a drug effect on the DAT occupancy specifically located in the VS (*P* < 0.05, paired-t-test) was found. BP values were not significantly modulated by the MPH in other striatal territories (*P* > 0.05). This result contrasted with the more widespread effect on DAT occupancy observed with high-dose MPH across all striatal territories, including antero-posterior and ventro-dorsal parts (P < 0.05). Together, this suggests that the therapeutic effect of low-dose MPH on impulsive decisions is associated with preferential action on DAT in the VS.

### Selective role of the ventral striatum in reducing impulsive choice

**Figure 4:**
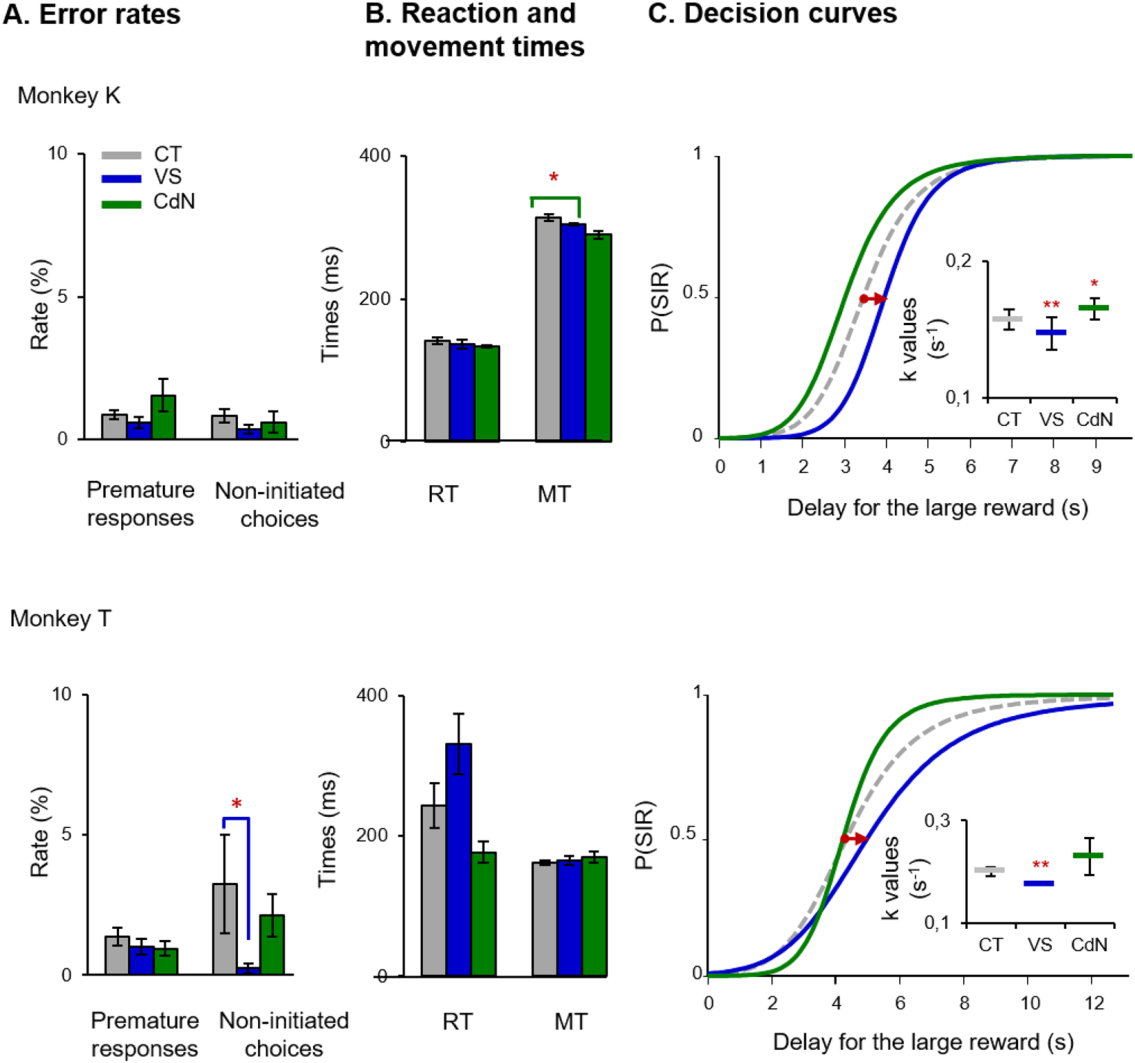
Delay discounting behaviours after intrastriatal administration of MPH. (**A**) Error rates show a decrease in non-initiated choices in monkey T after MPH micro-injections in the VS. (**B**) Reaction times were not altered by local administration, while MPH micro-injections in the CdN reduced movement times in monkey K (Mann-Whitney U-test; *P* > 0.05). (**C**) MPH micro-injections into the striatum were alternatively tested in different territories. Following MPH injections in the VS, there was a significant decrease in discount factor *k* for both monkeys (two-way ANOVA; ***P < 0.001), while no changes were detected for the CdN (*P* > 0.05). These figures follow the conventions of Fig. 2.

To determine whether the reduction in impulsivity triggered by low-dose MPH was selectively supported by one of the anterior striatal territories, intracerebral MPH administration was performed on two monkeys. Micro-injections were performed alternatively in the right VS or CdN at the beginning of different sessions (Fig. 4). Each striatal subregion was tested with the drug during four experimental sessions per monkey, and analyses were limited to early periods in order to minimize the effects due to diffusion into other territories. Figure 4 illustrates selective behavioural changes obtained after distinct MPH micro-injections. Unlike with intramuscular administration, no consistent impairments in animal performance, such as an alteration of error rates (Fig. 4A), RTs or MTs (Fig. 4B), were found. Task performance tended to be partially improved, with faster movements for monkey K (*P* = 0.01 Mann-Whitney U-test) and fewer omitted trials for monkey T (*P* = 0.02), but these drug effects were triggered in different striatal territories.

Importantly, we found that only one subregion in the striatum supported MPH action on temporal discounting (Fig. 4C). MPH micro-injections in the VS showed a consistent reduction in *k* values (*F* > 5.19, *P* < 0.01), while administration in the CdN impacted impulsive choices in monkey K (*F* = 8.80; *P* > 0.01), increasing them (see also the detailed results in supplementary information). Thus, following MPH micro-injections in the VS, monkeys K and T showed fewer impulsive choices, with decreases of 12% and 5% in their temporal discount rates, respectively. Because the strength of the drug effect on temporal discounting was equivalent between local and systemic MPH injections (P > 0.05, Mann-Whitney U-test), it is quite likely that the VS constitutes one of the main action sites reached by MPH to therapeutically reduce impulsive choices.

## Discussion

Here, we aimed to determine which striatum territory, the VS or CdN, is involved in reducing impulsive choices triggered by MPH, taking into account the dose effect of this drug. Using intramuscular injections, we found that low-dose MPH reduced the discounting factor on the DDT, while high-dose MPH had adverse effects on the animals, inducing a sharp increase in omissions and making them unable to perform the task. On PET scans, we observed that low-dose MPH induced a statistically significant occupancy of the DAT in both ventral striatal territories (the anterior VS and the posterior-ventral putamen), while high-dose MPH blocked dopamine reuptake in the entire striatum, including the ventral and dorsal parts. Then, using MPH micro-injections in the anterior striatum, we showed that the VS was the only territory to reduce impulsive choices.

Our results on dose effect are consistent with the literature, as sedation is one of the main side effects reported in patients with ADHD^17^. Clatworthy *et al*. formulated a strong hypothesis suggesting an inverted U-shaped relationship between cognitive performance and dopaminergic activity^43^. The increase in intrastriatal dopamine rate is believed to be beneficial at a low dose, but detrimental at a high dose. In our study, we observed a strong increase in non-initiated choices or omissions at the high dose, suggesting a decrease in motivation or a general sedative effect, which may result from MPH action on different processes, such as visual perception from the ventral-posterior striatum^44,45^, attention disturbance from the anterior and posterior CdN^46,47^ and movement initiation from the Putamen, which could be under the control of dopamine modulation, as supported by our high-dose PET imaging results. Moreover, noradrenergic modulation at the cortical level^48^ could not be excluded either. Because the MPH intramuscular injection is a general and nonselective administration method, these various effects could result from different action sites reached by the drug.

Consistent with the literature^9–14^, therapeutic effects on impulsive choices were observed at the low dose. Notably, the MPH effect depended on the animals’ initial impulsive state^49,50^. Here, the *k* discount factor allowed us to characterize our monkeys’ phenotype. The strongest effect was observed in monkey A, which had the highest basic discount factor. On the contrary, we found no reduction in the discounting factor of monkey T, which was the least impulsive monkey. Thus, at our dose, MPH seemed to be effective only on highly impulsive phenotypes. These behavioural results obtained in three monkeys performing the DDT are consistent with previous studies in rodents showing that MPH had different effects depending on the animals’ basic phenotype^49,50^.

On PET imaging, low-dose MPH induced significant DAT occupancy, specifically in the two ventral striatum territories (anterior and posterior). Based on the fact that the anterior VS is involved in motivation^51,52^ and encodes subjective reward value^23^, whereas the ventral posterior putamen is involved in visual perception processes^44^, we hypothesized that the beneficial effect of MPH is mediated by the anterior VS. This hypothesis was confirmed with intrastriatal micro-injections. Indeed, MPH micro-injections in the VS induced a decrease in the discounting factor in monkeys. This is consistent with the fact that the VS, and particularly the central region that we injected in, is involved in reward expectation^51,52^ as well as in decision-making processes based on negative values^53^, in aversive anticipation^54^ and in anxiety-related behaviors^26,55^ such as active avoidance^56,57^. Therefore, MPH is believed to reduce the aversive impact of delayed reward by acting directly on the temporal devaluation encoded specifically in the central region of the VS that also controls aversive avoidance^53^. In other words, MPH in the VS makes animals more patients in their choices by decreasing their delay aversion.

Within the anterior striatum, the CdN was also a region of interest regarding impulsive choices what led us to performed MPH micro-injections in this other striatal territory. Interestingly, when MPH was injected in the CdN, our monkeys tended to be more impulsive, choosing the SIR more often. This is consistent with our previous study, in which the same group of monkeys was more impulsive after pramipexole injections in the CdN^25^. Thus, the increase in dopamine effect, either by blocking reuptake or by acting with an agonist, can exert an opposite effect on impulsivity depending on the striatal territory on which the drug preferentially acts. This opposite effect of MPH in the VS and CdN may explain the lack of MPH effect induced by peripheral administration, as we observed in monkey T. The MPH injected directly into the striatal territories induced opposite effects, which can cancel each other during IM injections. From a clinical point of view, these latter results may also explain the variability in MPH effects between individuals and the lack of beneficial effects on some patients. These results also point out that dopamine modulators such as MPH or agonists as Pramipexole, can induce a wide variety of dose-dependent therapeutic or adverse effects, due to the heterogeneity and high levels of free dopaminergic receptors inside the different functional territories of the striatum. All of these results support the hypothesis that distinct cortico-striatal loops are involved in different facets of impulsivity^22^ and, in the case of decision impulsivity, the VS and the CdN are involved in opposite ways.

According to McClure *et al*.^58^, impulsive choices are processed by two separate systems in competition. They propose a limbic cortico-striatal system, including the VS, that processes reward valuation and produces SIR choice, whereas a cognitive system, including the lateral prefrontal cortex, is engaged in choices irrespective to delay^58^. This second system could involve the CdN, where we produced an increase in impulsive tendencies. Our results confirm the separate role of these two systems and support the hypothesis of MPH reducing the SIR valuation by attenuating aversion to delay, switching the balance in favour of the cognitive system. Together, these results show the specific role on dopamine depending on the striatal territory.

Notably, our results in healthy animals point in the same direction as the literature on ADHD patients. As Volkow *et al*. showed that ADHD patients present reductions in DAT and D_2/3_ receptor levels in the VS^59^, so we confirm that this structure plays a key role in the therapeutic effects of MPH on impulsivity.

Lastly, it appears important to summarize some limitations of this study. First, we used a version of the DDT without post-reward delays to equalize trial lengths. Hence, our procedure assessed the overall delay aversion during the task, without being able to dissociate the sensitivity for the delay occurring before the upcoming reward from the willingness to maximize the rate of reinforcement. Those possible confounding factors induces usually a systematic bias in measures of animal temporal discounting, even when post-reward delays are added in the task^60^. Second, although the density of norepinephrine transporters appears weak in the striatum compared to dopamine transporters^48^, we cannot exclude the possibility that the norepinephrine system contributes to MPH effects on task performance. Third, our monkey model did not present the pathophysiological underpinnings of ADHD patients, and our MPH administrations did not reproduce a usual chronic treatment. Those differences may potentially involve different neural mechanisms related to the beneficial effect of MPH.

Despite these limitations, our study filled the gap between rodents and humans, showing that therapeutic effects on impulsive choices induced by MPH are processed in the VS and adverse effects at high dose are probably due to activation other striatal territories. These results support the hypothesis that distinct cortico-striatal loops are involved in different aspects of impulsivity^22^. Finally, at the clinical level, the effects of MPH depend on the initial impulsive state of the animal, suggesting that dopamine modulation through the VS is central to reducing impulsive choices^61^.

## Supplementary information

Supplementary figure 1.
