# Supplementary material for: Ventral striatum supports Methylphenidate therapeutic effects on impulsive choices expressed in temporal discounting task
Source: Sci Rep. 2020 Jan 20;10:716. doi: 10.1038/s41598-020-57595-6 (PMC6971276; doi:10.1038/s41598-020-57595-6)
Supplement: Supplementary file 1 — Supplementary figure 1. [file 41598_2020_57595_MOESM1_ESM.docx]

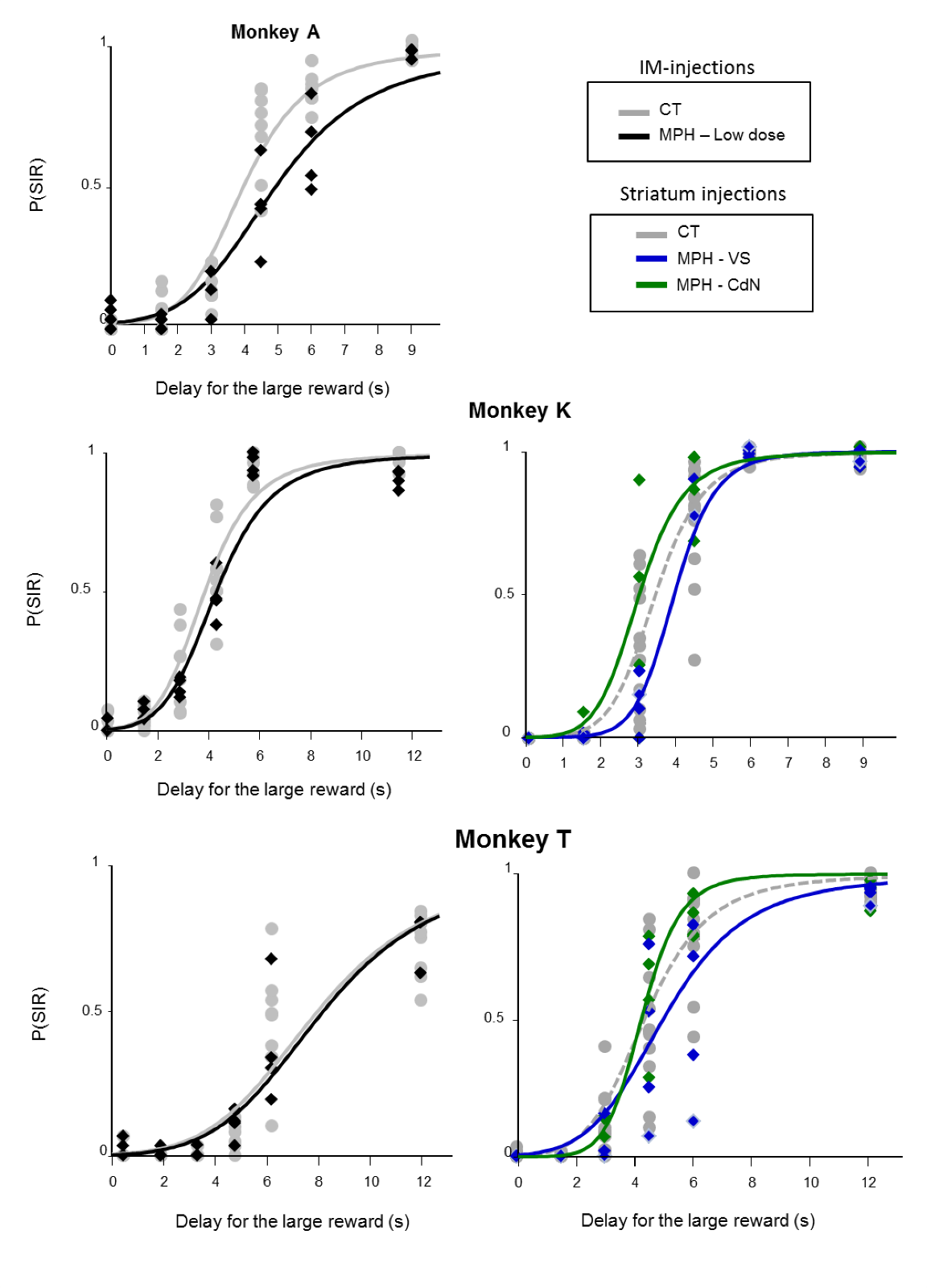


**Supplementary figure 1:** Decision curves with intramuscular (*left*) or intrastriatal (*right*) administrations of MPH for the three monkeys (A, K and T). These curves follow the conventions of figures 2 and 4. The dots represent ratios calculated for each combination of choice between the SIR and the LDR. The fitting logistic functions were plotted using exponential functions to estimate the temporal discounting behaviour with a maximum likelihood procedure (for details, see materials and methods).
